# Frontline Therapy in Diffuse Large B‐Cell Lymphoma: Evolving Standards, Biological Insights, and Future Directions

**DOI:** 10.1111/ejh.70129

**Published:** 2026-02-03

**Authors:** Mamdouh Skafi, Santino Caserta, Ernesto Vigna, Antonella Bruzzese, Nicola Amodio, Eugenio Lucia, Virginia Olivito, Caterina Labanca, Francesco Mendicino, Maria Eugenia Alvaro, Fortunato Morabito, Enrica Antonia Martino, Massimo Gentile

**Affiliations:** ^1^ Emergency and Internal Medicine Department Saint Joseph Hospital East Jerusalem Palestine; ^2^ Hematology Unit, Department of Onco‐Hematology AO of Cosenza Cosenza Italy; ^3^ Department of Experimental and Clinical Medicine University of Catanzaro Catanzaro Italy; ^4^ AIL Sezione di Cosenza Cosenza Italy; ^5^ Department of Pharmacy, Health and Nutritional Science University of Calabria Rende Italy

## Abstract

Diffuse large B‐cell lymphoma (DLBCL) remains the most common aggressive lymphoma, representing a biologically heterogeneous disease with diverse clinical behaviors. For more than two decades, R‐CHOP has been the cornerstone of frontline treatment, curing approximately two‐thirds of patients. Despite this success, a substantial subset—particularly those with high‐risk biology, double‐hit genetics, activated B‐cell‐like (ABC) subtype, or adverse clinical features—still experience relapse or refractory disease. Recent advances in lymphoma biology, immunotherapy, and targeted therapy have stimulated intense interest in improving frontline outcomes. Strategies include optimizing cytotoxic backbone regimens, incorporating antibody–drug conjugates (ADCs), immunomodulatory agents (IMiDs), bispecific antibodies, and exploring the feasibility of frontline CAR‐T cell therapy. This review provides a comprehensive and discursive synthesis of the biological rationale, clinical evidence, trial results, and practical considerations shaping contemporary frontline treatment. We highlight the emerging role of molecular subtyping, the tumor microenvironment, and high‐risk biomarkers, while discussing ongoing challenges and opportunities in integrating novel modalities into standard practice. Although R‐CHOP remains the universal backbone, the therapeutic landscape is entering a transformative era, with polatuzumab‐based regimens, bispecific combinations, and precision‐guided approaches positioned to redefine frontline care for selected subgroups.

## 1 Introduction

Diffuse large B‐cell lymphoma (DLBCL) is the most common aggressive non‐Hodgkin lymphoma (NHL), representing approximately 30%–40% of adult NHL cases across Western and Asian populations, with an annual incidence of 5–7 per 100 000 individuals [1, 2].

Historically, the introduction of the CHOP regimen (cyclophosphamide, doxorubicin, vincristine, prednisone) marked a turning point in the management of aggressive lymphomas, substantially improving survival. The later addition of the anti‐CD20 antibody rituximab to CHOP—yielding the now‐canonical R‐CHOP regimen—further enhanced outcomes, and established R‐CHOP as the global frontline standard for DLBCL for decades [3, 4].

Despite its success, R‐CHOP fails to cure approximately 30%–40% of patients, who ultimately experience refractory disease or relapse, often with poor outcomes upon salvage therapy [1, 5, 6, 7].

Several factors contribute to treatment failure. First, biological heterogeneity has profound prognostic implications [6, 8]. Outcomes are particularly poor in specific molecular subgroups, including activated B‐cell‐like (ABC) DLBCL—driven by chronic active B‐cell receptor (BCR) and NF‐κB signaling—high‐grade B‐cell lymphomas with MYC and BCL2/BCL6 rearrangements (double‐hit or triple‐hit lymphomas), and double‐expressor lymphomas (DEL), which coexpress MYC and BCL2 proteins without underlying rearrangements. Foundational genomic studies from the NCI and others have detailed the pathogenesis and therapeutic vulnerabilities of these entities [9, 10]. Consistently inferior outcomes with standard R‐CHOP have been reported in these biologically defined groups, motivating the pursuit of alternative frontline strategies [11].

Second, high‐risk clinical features—advanced stage, bulky disease, elevated IPI or NCCN‐IPI scores—further worsen prognosis even in the rituximab era. Third, patient‐related factors, including advanced age, poor performance status, and comorbidities, may limit the safe delivery of anthracycline‐based chemoimmunotherapy. Finally, for patients who relapse or are refractory to first‐line therapy, outcomes have traditionally been dismal; historical median overall survival after primary refractory disease has been measured in months [6, 7].

Because of these limitations, a central question has persisted for nearly two decades: can we truly improve upon R‐CHOP?

Driven by advances in genomics, transcriptomics, and immunotherapy, the frontline therapeutic landscape for DLBCL is undergoing rapid evolution. New agents—including antibody–drug conjugates, bispecific antibodies, CAR‐T cell therapy, targeted inhibitors, and immunomodulatory agents (IMiDs)—are being tested to improve cure rates while maintaining tolerability.

This review aims to provide a comprehensive, integrative synthesis of the evolving landscape of first‐line therapy in DLBCL—linking biological rationale, translational insights, clinical evidence, and real‐world considerations—to outline how emerging strategies may be selectively incorporated into clinical practice in the years ahead.

## 2 Initial Attempts to Optimize Frontline R‐CHOP: Lessons Learned

Efforts to improve first‐line therapy for previously untreated DLBCL have historically followed three major strategies: modifying the monoclonal antibody component, intensifying or reconfiguring the chemotherapy backbone, and administering standard R‐CHOP in a dose‐dense schedule. These strategies were evaluated in several large randomized trials.

Two pivotal phase III studies exemplify the first two approaches. The GOYA trial (*n* = 1418) evaluated whether substituting rituximab with obinutuzumab (G‐CHOP vs. R‐CHOP) could improve outcomes [12], while CALGB 50303 (*n* = 491) tested chemotherapy intensification using dose‐adjusted EPOCH‐R (DA‐EPOCH‐R) versus standard R‐CHOP [13]. Neither trial demonstrated an improvement in progression‐free survival (PFS). In GOYA, the PFS hazard ratio (HR) was 0.92 (95% CI 0.76–1.11; *p* = 0.39) with similar 3‐year PFS rates (70% vs. 67%). In CALGB 50303, DA‐EPOCH‐R yielded an HR of 0.93 (95% CI 0.68–1.27; *p* = 0.65), with nearly identical 2‐year PFS (78.9% vs. 75.5%). Both alternative regimens produced more toxicity without efficacy gains: G‐CHOP resulted in higher rates of grade 3–5 events (73.7% vs. 64.7%) and serious adverse events (42.6% vs. 37.6%), while DA‐EPOCH‐R caused more febrile neutropenia (35.0% vs. 17.7%) and neuropathy (18.6% vs. 3.3%).

A third strategy to improve frontline outcomes in DLBCL—the adoption of a dose‐dense R‐CHOP schedule administered every 14 days (R‐CHOP‐14) instead of the conventional 21‐day interval (R‐CHOP‐21)—has been rigorously evaluated in multiple randomized studies. The pivotal UK NCRI R‐CHOP14v21 phase III trial, a large, contemporary study, demonstrated no improvement in progression‐free or overall survival with dose‐dense therapy, effectively challenging the rationale for 14‐day scheduling in unselected patients [14]. A dedicated analysis of elderly patients from the same trial similarly showed no clinical advantage and a higher burden of hematologic toxicity with R‐CHOP‐14 (PMID: 25605933). Earlier studies from the German GELA/RICOVER program—while instrumental in establishing the benefit of adding rituximab—did not provide definitive evidence that the 14‐day schedule itself was superior; later long‐term follow‐up of the R‐CHOP14 versus R‐CHOP21 comparison (particularly the LNH03‐6B study) confirmed the absence of sustained differences in PFS or OS between the two approaches [15]. Finally, a meta‐analysis of randomized trials corroborated these findings, showing no survival benefit for dose‐dense therapy and a higher incidence of toxicity [16].

Real‐world evidence corroborates the findings of randomized trials, demonstrating no survival advantage for dose‐dense R‐CHOP‐14 over standard R‐CHOP‐21. The prospective German Tumour Registry Lymphatic Neoplasms analyzed 582 patients treated in routine practice across 92 centers, with approximately 45% receiving R‐CHOP‐14 and 55% R‐CHOP‐21. Three‐year overall survival was virtually identical between the two schedules (84% for both arms) and remained similar among patients who completed the first 4 cycles according to their initial schedule (87% vs. 89%) [17]. Importantly, the registry also highlighted a higher incidence of hematologic toxicity and need for growth factor support with the 14‐day schedule, emphasizing the unfavorable risk–benefit profile of dose‐dense R‐CHOP outside of clinical trials.

The reproducibility of these negative findings across antibody substitution, chemotherapy intensification, and dose‐dense administration underscores how difficult it is to surpass R‐CHOP in an unselected population. Moreover, these studies highlighted the importance of disease biology: CALGB 50303 noted unexpectedly strong R‐CHOP outcomes relative to historical cohorts, possibly reflecting trial selection bias, while GOYA showed that cell‐of‐origin (GCB vs. ABC) influenced prognosis independently of treatment choice.

Table 1 summarizes the major randomized attempts to improve frontline R‐CHOP through antibody substitution, chemotherapy intensification, and dose‐dense scheduling. Although each strategy was supported by a strong biological rationale, none resulted in superior progression‐free or overall survival, and several were associated with increased toxicity. Together, these insights reinforce that future progress will depend on biomarker‐driven, mechanism‐based strategies tailored to molecular or clinical subgroups.

**TABLE 1:** Major initial attempts to improve frontline R‐CHOP in DLBCL.

Strategy	Trial	Design/comparison	Efficacy results	Toxicity profile	Conclusion
Antibody substitution	GOYA *N* = 1418	Obinutuzumab‐CHOP (G‐CHOP) vs. R‐CHOP	No PFS benefit: HR 0.92 (95% CI 0.76–1.11, *p* = 0.39); 3‐year PFS 70% vs. 67%	Higher grade 3–5 AEs (73.7% vs. 64.7%); more SAEs (42.6% vs. 37.6%)	G‐CHOP increased toxicity with no clinical benefit
Chemotherapy intensification	CALGB 50303 (Alliance) *N* = 491	DA‐EPOCH‐R vs. R‐CHOP	No PFS benefit: HR 0.93 (95% CI 0.68–1.27, *p* = 0.65); 2‐year PFS 78.9% vs. 75.5%	More febrile neutropenia (35% vs. 17.7%) and neuropathy	DA‐EPOCH‐R more toxic, no advantage; R‐CHOP remains standard
Dose‐dense R‐CHOP	UK NCRI R‐CHOP14v21 *N* > 1000	R‐CHOP‐14 vs. R‐CHOP‐21	OS and PFS identical; no advantage in elderly subgroup	More hematologic toxicity with 14‐day schedule	Dose‐dense not superior; 21‐day schedule preferred
Dose‐dense (supporting evidence)	German RICOVER/LNH03‐6B *N* = Several thousand patients across programs	CHOP‐14 ± *R* vs. CHOP‐21 ± *R*	No long‐term PFS/OS benefit of 14‐day vs. 21‐day	More G‐CSF use, higher myelosuppression	Confirms lack of benefit of dose‐dense schedule
Dose‐dense (real‐world)	German TLN Registry *N* = 582 (routine practice)	R‐CHOP‐14 vs. R‐CHOP‐21	3‐Year OS identical: 84% both arms; similar OS by on‐protocol analysis	Higher hematologic toxicity and G‐CSF use with R‐CHOP‐14	Real‐world practice confirms no clinical advantage

## 3 Targeting Immune and Oncogenic Pathways in First‐Line DLBCL: Lenalidomide and BTK Inhibitors

Advances in the molecular understanding of DLBCL have demonstrated that this disease comprises multiple biologically distinct subtypes with divergent genetic programs, signaling dependencies, and microenvironmental interactions [18]. Early gene‐expression profiling revealed that the ABC subtype is driven by chronic BCR signaling and constitutive NF‐κB activation, mechanisms associated with inferior outcomes under standard therapy [19, 20]. Genomic studies subsequently identified recurrent mutations in components of the BCR–NF‐κB pathway (such as *CD79A/B*, *MYD88*) that reinforce this signaling dependency, while dysfunction in antigen presentation and immune synapse formation has been implicated in immune evasion [21, 22].

This biological rationale spurred the development of frontline regimens that combine standard chemoimmunotherapy (R‐CHOP) with agents designed to restore immune competence and modify the tumor microenvironment (TME), or directly interrupt pathogenic signaling pathways essential to ABC–DLBCL survival. In this context, two of the most extensively studied strategies are: adding the immunomodulator Lenalidomide, and integrating inhibition of BCR signaling—for example, via the BTK inhibitor ibrutinib.

The US intergroup phase II trial ECOG‐ACRIN E1412 randomized untreated DLBCL patients to *R* ^2^‐CHOP vs. R‐CHOP and reported a 34% reduction in risk of progression or death, with a 3‐year PFS of 73% versus 61% in favor of *R* ^2^‐CHOP [23]. The benefit was observed across molecular subtypes, though the trial was not powered for definitive subtype‐based conclusions.

Encouraging early‐phase data prompted the biomarker‐driven phase III ROBUST study, which enrolled only centrally confirmed ABC‐type DLBCL patients. Surprisingly, the addition of lenalidomide at 15 mg/day (Days 1–14 of each 21‐day cycle) to R‐CHOP did not significantly improve PFS (HR 0.85, *p* = 0.29), and grade 3–4 hematologic toxicity was more frequent in the *R* ^2^‐CHOP arm [24].

These discordant results between E1412 and ROBUST likely reflect key differences in trial design, patient risk profiles (E1412 included higher‐risk patients), lenalidomide dosing, and possible differences in compliance and dose intensity—factors critical in chemoimmunotherapy combinations [24].

Real‐world evidence on *R* ^2^‐CHOP, or studies directly comparing this regimen with standard R‐CHOP, is currently lacking [25].

Overall, while lenalidomide cannot be recommended as standard frontline therapy, it may still hold value in carefully selected patients—particularly older or frail individuals and those treated within immunotherapy‐based combination strategies. Its immunomodulatory profile positions lenalidomide as a potentially meaningful adjunct in future biology‐driven, personalized approaches to frontline DLBCL.

Given the central role of BCR–NF‐κB signaling in ABC–DLBCL, targeting this pathway with BTK inhibitors has a strong mechanistic basis. Early preclinical and relapsed/refractory clinical data confirmed that ABC but not germinal center B‐cell‐like (GCB) DLBCL cell lines are sensitive to BTK inhibition [19, 20].

This led to the PHOENIX randomized phase III trial, which added ibrutinib 560 mg/day to R‐CHOP in untreated non‐GCB DLBCL. In the overall intent‐to‐treat population, ibrutinib did not significantly improve outcomes; however, subgroup analyses revealed that patients younger than 60 years old experienced substantial benefit—particularly those with genetically defined subtypes (“MCD” or “N1”) harboring *MYD88*/*CD79B* mutations—with markedly improved event‐free survival [26, 27].

These findings underscore that the therapeutic potential of BTK inhibition is likely confined to biologically defined, molecularly selected subgroups rather than the broader non‐GCB population.

In sum, the clinical experience with both lenalidomide and BTK inhibitors added to R‐CHOP highlights the challenges of translating biological rationale into broadly efficacious first‐line regimens for DLBCL. While early‐phase studies and subgroup signals are encouraging, definitive randomized data in unselected or broadly defined biomarker‐selected populations have largely failed to show consistent benefit. The marked heterogeneity of DLBCL—in genetics, signaling dependencies, immune microenvironment, and host factors (age, comorbidity, ability to tolerate intensive therapy)—is the main obstacle to broad success.

These observations strongly support a paradigm shift toward precision‐based, biomarker‐driven therapy in DLBCL: future trials should prospectively stratify by genetic subtype, signaling addiction (e.g., BCR/NF‐κB), microenvironmental features, and host factors—rather than relying on broad, nondiscriminatory combinations. Only through such tailored approaches will it be possible to deliver the right biological intervention to the right patient, maximizing benefit while minimizing unnecessary toxicity.

Importantly, clinical outcomes and biological mechanisms associated with rituximab‐based combinations are not interchangeable across different hematologic malignancies, nor are they uniformly applicable across biologically distinct subgroups within DLBCL. In particular, combinations of rituximab with novel agents—such as lenalidomide, Bruton tyrosine kinase inhibitors, or antibody–drug conjugates—may display divergent efficacy and toxicity profiles depending on disease context, cell‐of‐origin, underlying genetic alterations, and features of the TME. Accordingly, therapeutic benefit and mechanistic insights observed in one lymphoma subtype, or even within a specific subgroup of DLBCL, should not be extrapolated indiscriminately to others. This biological and clinical heterogeneity underscores the need for subgroup‐specific interpretation of clinical trial results and supports a precision‐based approach to the development of frontline therapies in DLBCL.

## 4 Targeting High‐Risk DLBCL With Polatuzumab Vedotin

In DLBCL, the transmembrane CD79b protein—a component of the BCR complex—is ubiquitously expressed across the majority of mature B‐cell malignancies, making it an ideal target for ADC therapy. In a foundational translational study, Polatuzumab vedotin (the anti‐CD79 b ADC) was shown to bind human CD79b with high affinity, maintain plasma stability, and exert robust antitumor effects in preclinical xenograft models and in vitro B‐cell lymphoma lines [28]. Another key work demonstrated that anti‐CD79b–MMAE conjugates induced cell death across a broad panel of DLBCL cell lines—both ABC and germinal center B‐cell–like (GCB) subtypes—regardless of the presence of CD79B signaling mutations, supporting the notion that CD79b expression (rather than signaling pathway status) is the critical determinant of ADC sensitivity [29].

By coupling the cytotoxic payload monomethyl auristatin E (MMAE) to an anti‐CD79b antibody, Polatuzumab vedotin selectively delivers a potent microtubule inhibitor to malignant B cells while sparing non‐B‐cell tissues. This selective cytotoxicity exploits the high mitotic rate typical of aggressive, BCR‐driven DLBCL and may bypass mechanisms of anthracycline resistance.

Moreover, pharmacodynamic studies in nonhuman primates provided evidence of selective depletion of proliferating B cells after ADC administration, confirming the mechanism where ADC binding to CD79b initiates internalization, linker cleavage, release of the cytotoxic payload (monomethyl auristatin E, MMAE), and resultant mitotic arrest and apoptosis—with favorable pharmacokinetics and manageable toxicity [30].

Finally, early‐phase clinical data in relapsed or refractory B‐cell NHL, including DLBCL, demonstrated that Polatuzumab vedotin was tolerable at an identified phase II dose, with signs of single‐agent activity and an acceptable safety profile, supporting its transition into combination and frontline trials [31].

Together, these preclinical and translational findings provide a compelling biological rationale for using CD79b‐directed ADC therapy in DLBCL. They underpin the decision to replace—or refine—components of conventional chemoimmunotherapy (e.g., vincristine in CHOP) with targeted ADCs such as Polatuzumab vedotin, especially in high‐risk or biologically aggressive subtypes, where standard regimens may be suboptimal.

The pivotal phase III POLARIX trial evaluated Polatuzumab vedotin combined with rituximab, cyclophosphamide, doxorubicin, and prednisone (Pola‐R‐CHP), replacing vincristine in the CHOP backbone. The trial enrolled previously untreated intermediate‐ to high‐risk DLBCL patients and demonstrated a statistically significant improvement in PFS compared with standard R‐CHOP. At a median follow‐up of 28 months, 2‐year PFS was 76.7% versus 70.2% (HR 0.73; 95% CI 0.57–0.95; *p* = 0.02), without a meaningful increase in grade ≥ 3 adverse events, indicating a favorable risk–benefit profile [32]. Subgroup analyses suggested particular benefit in patients with ABC‐type disease, higher International Prognostic Index (IPI) scores, and more proliferative tumors. By substituting vincristine with Polatuzumab vedotin, Pola‐R‐CHP avoids overlapping neurotoxicity while adding targeted cytotoxic intensity, potentially benefiting older adults or those with baseline comorbidities.

Following approval of Polatuzumab vedotin in combination with rituximab, cyclophosphamide, doxorubicin, and prednisone (Pola‐R‐CHP) based on the pivotal POLARIX trial, early real‐world studies have begun to provide valuable insights into its effectiveness, safety, and feasibility in routine clinical practice. These studies are particularly important for understanding outcomes in patients often underrepresented in clinical trials, including the elderly, frail, or those with comorbidities.

A large multicenter retrospective study in China included 600 previously untreated DLBCL patients, with 131 receiving Pola‐R‐CHP and 469 R‐CHOP. After 1:2 propensity score matching, 128 matched pairs were analyzed. At a median follow‐up of 12.8 months, 12‐month PFS was numerically higher with Pola‐R‐CHP (90.3% vs. 84.1%; *p* = 0.18), and complete response (CR) rates favored Pola‐R‐CHP (86.8% vs. 79.7%; *p* = 0.09), though differences did not reach statistical significance. Safety profiles were comparable, and benefits were observed across molecular and clinical subgroups, including advanced stage, ECOG ≥ 2, extranodal involvement ≥ 2, and non‐GCB subtype. Correlations were observed between certain genotypes (e.g., PIM1, TP53) and treatment outcomes, supporting the biologic rationale for Pola‐R‐CHP in high‐risk molecular subgroups.

The Peking Union Medical College Hospital retrospective study compared 36 Pola‐R‐CHP patients to 36 matched R‐CHOP patients. Interim CR rates were 72.2% versus 63.9% (*p* = 0.035) and overall response rates (ORR) 100% versus 83.3% (*p* = 0.011) in favor of Pola‐R‐CHP. At a median follow‐up of ~13.3 months, there was one death and two progressions in the Pola group versus nine deaths and nine progressions in the R‐CHOP group. Twelve‐month OS was 97% versus 94%, and 12‐month PFS was 86% versus 94%; differences were not statistically significant, likely due to the small sample size. Importantly, safety profiles were comparable between groups.

Several studies have focused on elderly or frail patients. A multicenter Chinese cohort of 38 patients aged ≥ 80 years treated with reduced‐dose Pola‐R‐CHP reported 12‐month OS of 86.2% and PFS of 78.5%, with manageable hematologic toxicity and no neuropathy‐related dose reductions [33]. Japanese multicenter data similarly suggested that Pola‐R‐CHP is feasible and tolerable in octogenarians with DLBCL.

Additional analyses, including patients with comorbidities or lower IPI scores, further indicate that Pola‐R‐CHP maintains high response rates and an acceptable safety profile in broader, unselected populations [34].

Collectively, these real‐world data complement the POLARIX trial results, confirming that Pola‐R‐CHP can be effective and safe across diverse patient groups, including those with high‐risk disease, advanced age, or comorbidities. Limitations include short follow‐up, modest sample sizes, and low event rates, which preclude definitive conclusions on long‐term PFS, OS, or late toxicity. Nonetheless, these studies reinforce the biologic rationale for targeting CD79b in aggressive, BCR‐driven DLBCL and support the integration of Pola‐R‐CHP into first‐line therapy for high‐risk populations.

Building on clinical trial evidence, a recent network meta‐analysis encompassing 20 randomized controlled trials compared Pola‐R‐CHP with other novel first‐line therapies in previously untreated ABC‐type DLBCL. The analysis suggested superior progression‐free and overall survival with Pola‐R‐CHP, particularly in high‐risk, BCR‐driven disease. These findings reinforce the mechanistic rationale for targeting CD79b and support Pola‐R‐CHP as a preferred frontline option for patients with aggressive molecular features.

## 5 Bispecific Antibodies Entering First‐Line Therapy in DLBCL: Rationale and Emerging Landscape

At present, CD20 × CD3 bispecific antibody‐based regimens in the frontline treatment of DLBCL remain investigational and should be considered exclusively within the context of clinical trials.

In fact, the considerations discussed below are primarily based on early‐phase clinical trials and emerging evidence; therefore, interpretations regarding the potential role of bispecific antibodies in the frontline setting should be regarded as hypothesis‐generating and reflective of expert opinion rather than evidence‐based standard‐of‐care conclusions.

The TME critically shapes DLBCL biology, influencing disease progression, therapy response, and relapse risk. Beyond intrinsic B‐cell drivers, the composition and functional state of immune, stromal, and other non‐B‐cell elements are major determinants of outcome.

**FIGURE 1:**
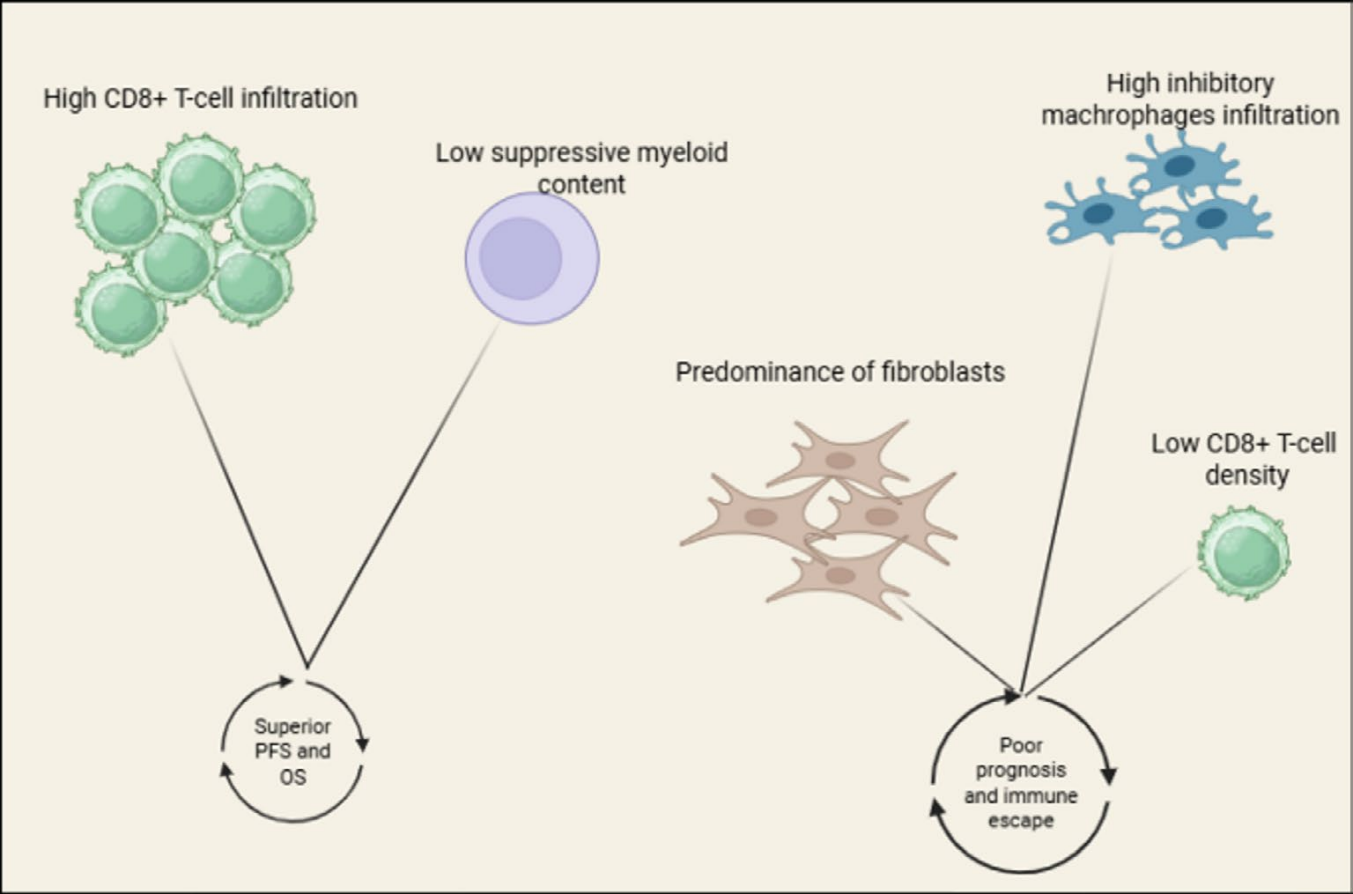
Immune‐hot vs. immune‐cold tumor microenvironments. Immune‐hot TMEs display dense infiltration of cytotoxic CD8^+^ T cells, low levels of suppressive myeloid cells, and an overall activated immune milieu, which collectively associate with improved progression‐free and overall survival. In contrast, immune‐cold TMEs are defined by scarce T‐cell presence, increased immunosuppressive macrophages, and a fibroblast‐dominant stroma, features linked to poor prognosis, enhanced immune escape, and reduced responsiveness to therapy. Created in BioRender. Santino Caserta (2025). HTTPS://APP.BIORENDER.COM/ILLUSTRATIONS/6935B7DCF0FCFEA3F2BDC7DE.

Immune‐hot TMEs—characterized by high CD8^+^ T‐cell infiltration and low suppressive myeloid content—are associated with superior progression‐free and overall survival [35, 36]. In contrast, TMEs with low T‐cell density, enrichment of inhibitory macrophages (e.g., PD‐L1^+^ or TIM3^+^ TAMs), or predominance of suppressive stromal/fibroblast elements correlate with poor prognosis and immune escape [37] (Figure 1).

Macrophage plasticity further complicates the TME: TAMs in DLBCL are heterogeneous, often exhibiting noncanonical phenotypes rather than classical M1/M2 polarization, reflecting diverse immunomodulatory roles. Stromal components—including fibroblasts and extracellular matrix—modulate angiogenesis, immune cell trafficking, and spatial organization of tumor–immune interactions, affecting chemosensitivity and immunotherapy response [38, 39].

These insights support the rationale for introducing CD20 × CD3 bispecific antibodies earlier in therapy. By simultaneously engaging T cells and B cells, bispecifics can bypass dysfunctional endogenous immunity, redirecting T‐cell cytotoxicity against malignant B cells irrespective of baseline T‐cell function or TME suppression. Preclinical and early clinical studies show activity across molecular subtypes and potential to overcome high‐risk TME features [40].

Frontline evaluation of bispecific antibodies reflects a conceptual shift: instead of relying on the existing immune contexture, these agents actively remodel the TME by directly activating T cells and establishing potent immune synapses. Consequently, bispecific antibodies represent a mechanistically compelling strategy to improve cure rates in patients with microenvironmental features predictive of poor response to standard chemoimmunotherapy.

In recent years, CD20 × CD3 bispecific antibodies have moved from the salvage setting toward first‐line therapy in DLBCL, initially through early‐phase combination studies and now via large randomized trials. R‐CHOP remains the standard initial regimen, but relapse in up to 40% of patients has driven exploration of whether adding T‐cell‐engaging antibodies can deepen responses from the outset [41].

The most mature first‐line experience comes from epcoritamab combined with R‐CHOP in high‐risk newly diagnosed patients within the EPCORE NHL‐2 trial (arm 1, NCT04663347). In this phase 1b/2 program, adults with previously untreated CD20‐positive DLBCL and an International Prognostic Index (IPI) of at least three received subcutaneous epcoritamab together with six 21‐day cycles of standard R‐CHOP, followed by epcoritamab monotherapy for up to 1 year. Step‐up dosing and corticosteroid prophylaxis were used during the first cycles to limit cytokine release syndrome (CRS), mirroring the mitigation strategy established in the relapsed setting. Updated phase 1/2 results showed that this fixed‐duration epcoritamab–R‐CHOP regimen induced high CR rates in patients with high‐risk features, including double‐ or triple‐hit biology, with a manageable toxicity profile [42]. CRS events were frequent but predominantly low‐grade: in one dataset, any‐grade CRS occurred in 45% of patients, with Grade 3 events in only 3%, all resolving with standard management, and without treatment discontinuation due to CRS. Longer follow‐up suggests that outcomes compare favorably with historical R‐CHOP alone in this high‐risk population and has directly supported a definitive randomized phase 3 trial, EPCORE DLBCL‐2 (NCT05578976), which is currently ongoing and compares epcoritamab plus R‐CHOP with R‐CHOP‐based standard of care in previously untreated DLBCL.

Mosunetuzumab has followed a slightly different developmental path but has also reached the frontline. In the phase 2 GO40515 study (NCT03677141), a dose‐escalation phase in relapsed or refractory B‐cell lymphoma was followed by a fixed‐dose expansion cohort in previously untreated DLBCL receiving mosunetuzumab plus CHOP (M‐CHOP) [43]. In the expansion phase, 40 newly diagnosed patients, more than half with IPI ≥ 3, received 6 cycles of intravenous mosunetuzumab together with CHOP. The final phase 2 analysis showed an ORR of 87.5% and a CR rate of 85.0%, with a toxicity profile dominated by neutropenia (70%), mostly in Cycle 1 and of short duration. CRS and neurologic events were generally low‐grade and manageable, reinforcing the feasibility of combining a BsAb with full‐dose CHOP in the first‐line setting. The same trial has also evaluated Mosunetuzumab in combination with polatuzumab‐CHP (Pola‐M‐CHP) and compared it with Pola‐R‐CHP in untreated DLBCL; in this randomized phase 1b/2 portion (still under the umbrella of NCT03677141), Pola‐M‐CHP achieved similar response rates to Pola‐R‐CHP, but in this relatively small cohort it did not demonstrate a clear clinical advantage over the rituximab‐containing regimen [44]. Thus, for mosunetuzumab, the phase 2M‐CHOP results can be regarded as a completed proof‐of‐concept experience, whereas further front‐line development is currently proceeding mainly through combinations and comparative studies embedded within NCT03677141 rather than through a separate large phase 3 trial.

Glofitamab, which has a 2:1 CD20:CD3 binding configuration and requires obinutuzumab pre‐treatment to limit CRS, was first established in heavily pretreated DLBCL in the pivotal phase 1/2 trial NCT03075696, where fixed‐duration monotherapy produced durable complete remissions in a fraction of patients [45]. Building on these results, several frontline approaches with glofitamab have emerged. One completed early‐phase experience comes from the NP40126 phase Ib study (NCT03467373), which includes a safety run‐in cohort testing glofitamab plus R‐CHOP in previously untreated DLBCL. In this ongoing but early‐phase trial, preliminary data from the 1 L safety run‐in showed that full‐dose R‐CHOP could be maintained, with a very low incidence of CRS and no neurotoxicity, suggesting that glofitamab–R‐CHOP is feasible even in the outpatient setting.

More mature front‐line data for glofitamab now come from the investigator‐initiated COALITION trial (NCT04914741), a randomized phase II study in patients aged 65 years or younger with high‐risk large B‐cell lymphoma [46]. All patients received one “pre‐phase” cycle of R‐CHOP and were then assigned to either 5 cycles of glofitamab plus Pola‐R‐CHP or 5 cycles of glofitamab plus R‐CHOP, followed by two consolidation cycles of glofitamab. Initial results show that both combinations are deliverable and produce high rates of durable response in this younger, high‐burden population, with acceptable safety, thereby supporting further exploration of glofitamab as part of first‐line chemo‐immunotherapy. COALITION is thus a completed randomized phase II signal‐generating trial, while larger confirmatory studies are still pending.

The most definitive glofitamab front‐line evaluation is now occurring in the ongoing randomized phase III trial NCT06047080. This study compares glofitamab in combination with polatuzumab‐R‐CHP against polatuzumab‐R‐CHP alone in previously untreated DLBCL and will test whether the addition of a CD20 × CD3 BsAb to a modern antibody–drug‐conjugate regimen can meaningfully improve outcomes [6]. In parallel, epcoritamab's role in the front line is being formally assessed in EPCORE DLBCL‐2 (NCT05578976), another phase III randomized trial that pits epcoritamab plus R‐CHOP against R‐CHOP–based standard care. Both NCT06047080 and NCT05578976 are ongoing and specifically designed to answer whether BsAb‐containing regimens should replace R‐CHOP alone in broad first‐line practice.

Taken together, these data show a clear developmental progression. Completed early‐phase studies such as EPCORE NHL‐2 arm 1 (NCT04663347) and GO40515/M‐CHOP (NCT03677141) have already demonstrated that adding CD20 × CD3 bispecific antibodies to standard CHOP‐based regimens in newly diagnosed DLBCL is feasible, with high CR rates and generally manageable CRS and myelosuppression [43]. Randomized phase II work, such as the COALITION trial (NCT04914741), has extended this concept into comparative, high‐risk populations, again with encouraging efficacy. The field has now entered the pivotal stage with ongoing phase III trials—EPCORE DLBCL‐2 (NCT05578976) for epcoritamab and NCT06047080 for glofitamab—that will determine whether these combinations can truly displace traditional R‐CHOP‐based therapy as the new first‐line standard.

Table 2 summarizes the progressive evolution of CD20 × CD3 bispecific antibodies from early feasibility studies to pivotal randomized trials in previously untreated DLBCL.

**TABLE 2:** Completed (A) and ongoing (B) first‐line CD20 × CD3 bispecific antibody trials in diffuse large B‐cell lymphoma.

A. Key completed phase 1/2 “pathfinder” bispecific trials in first‐line DLBCL				
Regimen (frontline)	Trial/ID	Phase/design	Population (1L)	Main message
Epcoritamab + *R*‐CHOP (fixed duration + epcoritamab maintenance)	EPCORE NHL‐2 (arm 1)—NCT04663347	Phase 1b/2, multicohort, single‐arm	Newly diagnosed CD20+ DLBCL with IPI ≥ 3 and high‐risk features (including DHL/THL)	High CR rates with durable responses and manageable CRS in high‐risk 1L DLBCL; directly supported moving to EPCORE DLBCL‐2
Mosunetuzumab + CHOP (M‐CHOP)	GO40515—NCT03677141 (M‐CHOP expansion)	Phase 1b/2, open‐label, dose‐escalation then expansion	Forty previously untreated DLBCL; > 50% with IPI ≥ 3	ORR ~88%, CR ~85%, with mainly neutropenia; established feasibility of BsAb + full‐dose CHOP in 1L
Mosunetuzumab + Pola‐CHP (Pola‐M‐CHP) vs. Pola‐R‐CHP	NCT03677141 (Pola‐M‐CHP vs. Pola‐R‐CHP)	Phase 1b/2, randomized, controlled (small)	Previously untreated DLBCL	Pola‐M‐CHP was active but did not show clear clinical benefit over Pola‐R‐CHP; supports feasibility but not superiority in this cohort
Glofitamab + *R*‐CHOP (with obinutuzumab pre‐treatment)	NP40126—NCT03467373 (1L safety cohort)	Phase Ib, dose‐escalation with 1L safety run‐in	Previously untreated DLBCL (1L cohort within broader R/R NHL study)	Showed that full‐dose R‐CHOP can be delivered with glofitamab, with very low CRS and no neurotoxicity in 1L cohort
Glofitamab + *R*‐CHOP vs. glofitamab + Pola‐R‐CHP (after 1 prephase R‐CHOP)	COALITION—NCT04914741	Phase II, randomized, investigator‐initiated	Patients ≤ 65 years with high‐risk LBCL (IPI ≥ 3, high NCCN‐IPI and/or DHL/THL)	Both regimens were deliverable, with high and durable responses in younger, high‐risk 1L LBCL; provided the signal to move glofitamab combinations into phase 3 (SKYGLO)

B. Ongoing phase 3 bispecific trials in first‐line DLBCL/LBCL				
Regimen (frontline)	Trial/ID	Phase/design	Population (1L)	Status/role
Epcoritamab (SC) + R‐CHOP vs. R‐CHOP alone	EPCORE DLBCL‐2—NCT05578976	Phase 3, global, multicenter, open‐label, randomized	Newly diagnosed CD20+ DLBCL (de novo or transformed FL): DLBCL NOS, HGBL with MYC/BCL2/BCL6 rearrangements, T‐cell/histiocyte‐rich LBCL, EBV+ DLBCL NOS, FL grade 3B; ~900 patients worldwide	Pivotal trial testing whether epcoritamab + *R*‐CHOP improves outcomes vs. standard R‐CHOP; enrollment ongoing, no efficacy data yet
Glofitamab + Pola‐R‐CHP vs. Pola‐R‐CHP alone	SKYGLO/GO44145—NCT06047080	Phase 3, multicenter, randomized, open‐label	Previously untreated CD20+ LBCL (DLBCL NOS, HGBL) with IPI 2–5, age 18–80, ECOG 0–2; ≈1130 patients randomized 1:1	Pivotal trial testing whether adding glofitamab to Pola‐R‐CHP improves efficacy over Pola‐R‐CHP alone; recruitment ongoing

Abbreviations: 1L, first‐line; CHOP, cyclophosphamide, doxorubicin, vincristine, prednisone; CR, complete response; CRS, cytokine release syndrome; DLBCL, diffuse large B‐cell lymphoma; IPI, International Prognostic Index; LBCL, large B‐cell lymphoma; NCCN‐IPI, National Comprehensive Cancer Network‐International Prognostic Index; ORR, overall response rate; Pola‐CHP, polatuzumab vedotin + CHP; Pola‐R‐CHP, rituximab + Pola‐CHP; R‐CHOP, rituximab + CHOP; R/R, relapsed/refractory.

At present, BsAb‐containing regimens in the first‐line DLBCL setting remain investigational, anchored in phase 1/2 experiences such as EPCORE NHL‐2 (NCT04663347), GO40515/M‐CHOP and Pola‐M‐CHP (NCT03677141), and NP40126 (NCT03467373), and in a completed phase II randomized trial such as COALITION (NCT04914741). The definitive answer on routine use as first‐line therapy awaits the results of the ongoing phase III trials NCT05578976 and NCT06047080, which will show whether the promising biological rationale and early response data translate into superior long‐term survival and an acceptable safety balance compared with optimized R‐CHOP‐based standards.

Accordingly, despite encouraging early‐phase efficacy signals, bispecific antibody‐containing regimens are not yet part of routine frontline management and should be interpreted as investigational strategies pending results from ongoing phase III trials.

## 6 CAR T‐Cell Therapy as First‐Line Treatment in High‐Risk DLBCL

Frontline CAR T‐cell therapy in DLBCL should currently be regarded as an investigational strategy, confined to prospective clinical trials and not recommended for routine practice outside of study protocols.

The most important proof‐of‐concept is the phase 2 ZUMA‐12 trial, which evaluated axicabtagene ciloleucel (axi‐cel) in adults with newly diagnosed, high‐risk LBCL who showed an inadequate early response to first‐line chemoimmunotherapy [47]. ZUMA‐12 was a prospective, multicenter, single‐arm study in which patients first received 2 cycles of rituximab‐based induction (typically R‐CHOP or R‐EPOCH), followed by interim PET (PET2). Only those with PET‐positive disease (Deauville 4–5) and high‐risk features—either double/triple‐hit lymphoma or an IPI ≥ 3—proceeded to leukapheresis, lymphodepleting chemotherapy, and axi‐cel infusion [48]. It is important to emphasize that these findings derive from single‐arm and early‐phase studies; thus, current considerations regarding frontline CAR T‐cell therapy reflect expert opinion informed by preliminary evidence rather than definitive, evidence‐based recommendations.

All 40 treated patients met this stringent definition of high‐risk disease, with a roughly even distribution of HGBL (double/triple‐hit) and high‐risk LBCL defined by IPI ≥ 3. At a median follow‐up of 15.9 months, axi‐cel achieved an ORR of 89%, with a CR rate of 78%, and with 73% of patients maintaining an ongoing response [49]. These results are striking, given that all patients had high‐risk features and PET‐positive disease after only 2 cycles of induction, a group historically associated with a very high risk of early failure on continued chemoimmunotherapy. ZUMA‐12 thus showed that axi‐cel, delivered early in the disease course, can induce deep and apparently durable remissions in patients for whom standard therapy is already failing.

The safety profile in ZUMA‐12 was consistent with previous axi‐cel experience in relapsed/refractory LBCL. CAR T‐related toxicities such as CRS and immune effector cell‐associated neurotoxicity syndrome (ICANS) were observed but were generally manageable. In extended reports, grade ≥ 3 neurotoxicity occurred in a minority of patients and was reversible, and no deaths were attributed to CRS or neurotoxicity. Overall, axi‐cel “exhibited a high complete response rate and a manageable safety profile” as first‐line therapy in this setting. Hematologic toxicity and infections reflected both prior induction and lymphodepletion but did not prevent delivery of therapy. These data indicate that, in selected fit patients treated in experienced centers, first‐line axi‐cel is feasible and does not introduce unexpected toxicity.

To place ZUMA‐12 in context, external comparator analyses have applied its eligibility criteria to real‐world cohorts treated with intensive chemoimmunotherapy. In one such study, patients who would have met ZUMA‐12 criteria but received standard treatment had substantially inferior outcomes compared with ZUMA‐12, underlining the magnitude of benefit that early axi‐cel may confer in this high‐risk population. Although indirect, these comparisons reinforce the biological rationale for bringing CAR T‐cell therapy forward in patients with both adverse biology and dynamic evidence of early treatment failure.

The promising phase 2 results of ZUMA‐12 have led directly to the global phase 3 ZUMA‐23 trial, designed to define the role of axi‐cel as true first‐line therapy in high‐risk DLBCL. ZUMA‐23 (NCT05605899) is the first randomized phase 3 study of CAR T‐cell therapy as an initial regimen for any cancer. Approximately 300 adults with histologically confirmed high‐risk LBCL (DLBCL, HGBL, or transformed follicular/marginal zone lymphoma) and an IPI of 4–5 are being enrolled. All patients receive 1 cycle of rituximab‐based chemoimmunotherapy and are then randomized 1:1 either to continue standard of care (five additional cycles of R‐CHOP or DA‐EPOCH‐R) or to receive axi‐cel.

In the axi‐cel arm, patients undergo leukapheresis and receive R‐CHOP or DA‐EPOCH‐R as bridging therapy, followed by fludarabine/cyclophosphamide lymphodepletion and a single axi‐cel infusion at 2 × 10^6^ CAR T cells/kg. The primary endpoint is event‐free survival assessed by blinded central review, with key secondary endpoints including overall survival, PFS, safety, quality of life, and pharmacokinetics. The trial permits enrolment of patients with controlled HIV or hepatitis B/C, thereby broadening generalizability, but excludes primary CNS lymphoma. ZUMA‐23 is currently open and accruing, with no efficacy data yet available.

ZUMA‐23 will address the central clinical question left open by ZUMA‐12: does upfront axi‐cel meaningfully improve event‐free and overall survival compared with optimized chemoimmunotherapy alone in patients with high‐risk, newly diagnosed LBCL? If the trial shows a clear benefit with an acceptable toxicity and quality‐of‐life profile, axi‐cel may become a new standard for first‐line treatment of patients with IPI 4–5 disease, analogous to the way second‐line CAR T has supplanted autologous transplantation in early relapsed LBCL. If outcomes are similar, or toxicity and logistics prove limiting, CAR T‐cell therapy will likely remain reserved for relapsed or refractory disease [50].

In practice, several issues will shape the eventual role of first‐line CAR T. These include center experience and infrastructure, manufacturing capacity and turnaround time, cost‐effectiveness, and the availability of alternative strategies such as bispecific antibodies in high‐risk DLBCL. Ethically, exposing newly diagnosed patients to a complex therapy with potentially serious acute toxicities must be justified by a clear survival advantage over R‐CHOP‐based regimens, which remain curative for many even in higher‐risk categories. Risk‐adapted approaches—using clinical scores, biological markers, and early PET response—are therefore likely to be central to any frontline CAR T strategy.

At present, axi‐cel as first‐line therapy in DLBCL should be considered an investigational option confined to clinical trials such as ZUMA‐12 and ZUMA‐23. Together, these studies have established feasibility, signaled substantial efficacy in selected high‐risk patients, and will soon provide definitive comparative data against standard chemoimmunotherapy. Until ZUMA‐23 reports, R‐CHOP‐based regimens remain the standard of care, while first‐line CAR T‐cell therapy stands as a promising, but as yet unproven, strategy to improve outcomes in the highest‐risk DLBCL populations [51].

## 7 Conclusion

Frontline therapy for DLBCL is in a genuine transition, but R‐CHOP is not yet dethroned. Large randomized trials and contemporary reviews consistently show that empiric intensification or simple modification of R‐CHOP has not improved survival in unselected patients, underscoring its robustness as a backbone while highlighting the need for biologically guided innovation rather than more chemotherapy [1]. Polatuzumab‐vedotin‐based Pola‐R‐CHP is, at present, the most solidly validated step beyond classic R‐CHOP: the POLARIX trial demonstrated a significant PFS advantage in intermediate‐ and high‐risk patients without a major toxicity penalty, and subsequent analyses reinforce its mechanistic rationale in aggressive, BCR‐driven disease [32]. From an expert perspective, this justifies Pola‐R‐CHP as a preferred option for many high‐risk patients when accessible, while R‐CHOP remains entirely appropriate for standard‐risk disease and for health systems where cost or infrastructure limit access to novel agents.

Bispecific CD20 × CD3 antibodies and CAR T cells extend this evolution by directly exploiting or replacing host immunity. Early phase data with mosunetuzumab‐CHOP and other BsAb–chemo combinations show high CR rates and acceptable, largely manageable cytokine‐mediated toxicity in previously untreated, often high‐risk DLBCL, providing clear proof of concept for T‐cell‐engaging antibodies in the frontline setting [41]. Similarly, ZUMA‐12 demonstrates that axi‐cel delivered early in high‐risk, PET‐positive disease achieves deep and durable remissions with a safety profile consistent with later‐line use, suggesting a role for frontline CAR T in a narrow, biologically and dynamically selected subset if phase III data from ZUMA‐23 confirm a survival benefit [47]. For now, however, both bispecific‐containing regimens and upfront CAR T must be regarded as investigational in first line, appropriately confined to clinical trials.

More broadly, the heterogeneous results observed across frontline trials in DLBCL reflect several recurring methodological and biological challenges. Patient selection strongly influences outcomes, as trials enrolling unselected or clinically heterogeneous populations may dilute benefit signals confined to molecularly defined subgroups. Biomarker misclassification—particularly when based on surrogate assays for cell‐of‐origin or incomplete genomic profiling—can further obscure treatment effects and contribute to negative or inconclusive results. In addition, dosing intensity and treatment adherence are critical variables in chemoimmunotherapy combinations, especially in older or frail patients, where reduced dose intensity may offset potential therapeutic gains. The choice of trial endpoints also warrants careful consideration: PFS improvements may not uniformly translate into overall survival benefit, particularly in diseases with effective salvage options. Finally, the intrinsic biological heterogeneity of DLBCL, encompassing genetic alterations, signaling dependencies, immune microenvironmental features, and host‐related factors, limits the effectiveness of empiric treatment intensification across broad populations. Collectively, these considerations support a paradigm in which future advances in DLBCL will be driven by biomarker‐guided, risk‐adapted strategies rather than uniform escalation of frontline therapy.

**FIGURE 2:**
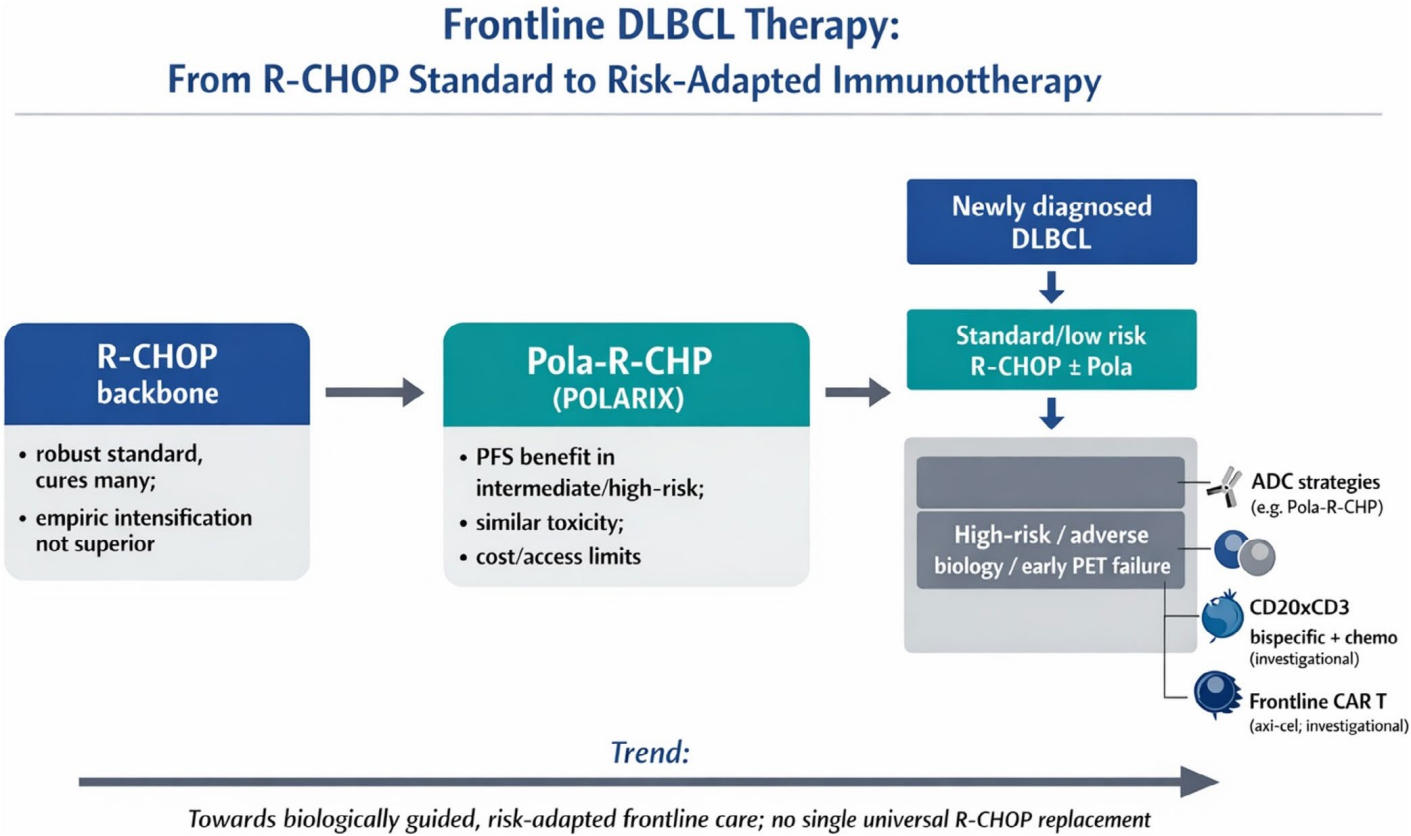
Evolving frontline treatment paradigm in diffuse large B‐cell lymphoma. The diagram summarizes how R‐CHOP remains the central, empirically validated backbone of frontline therapy, while polatuzumab vedotin‐based Pola‐R‐CHP represents the best‐validated incremental advance, particularly in intermediate‐ and high‐risk disease. On the right, a risk‐adapted biology‐driven pathway is depicted, in which patients with standard‐risk features continue to receive R‐CHOP (with or without polatuzumab), whereas those with adverse genomics, high clinical risk, or early PET‐defined failure are triaged toward more intensive immunologic strategies. Antibody–drug conjugate (ADC) approaches, CD20 × CD3 bispecific antibody–chemo combinations, and frontline CAR T‐cell therapy are shown as immunologic modalities under active investigation, emphasizing that the goal is not to replace R‐CHOP with a single universal regimen, but to individualize escalation beyond R‐CHOP based on biological risk and early response [1, 3, 4, 40, 41]. AI‐assisted tools (OpenAI, ChatGPT image generation) were used to support the creation of the schematic Figure 2. All content was reviewed and approved by the authors, who take full responsibility for the work.

Taken together, current evidence supports a near‐term paradigm in which frontline DLBCL therapy becomes explicitly risk‐adapted and biology‐driven. This emerging treatment framework is summarized in Figure 2, which schematically contrasts the enduring R‐CHOP backbone with polatuzumab‐based intensification and investigational immunologic strategies for biologically defined high‐risk subsets. R‐CHOP (with or without polatuzumab) will continue to cure many patients, while those with adverse genomics, high clinical risk, or early PET‐defined failure are triaged toward more intensive immunologic strategies within trials. The key challenge, in my view, is no longer to find a single universal “R‐CHOP replacement,” but to use genomic profiling, microenvironmental insights, and early response assessment—integrated with robust randomized data—to decide *who* truly needs more than R‐CHOP, what additional modality to deploy (ADC, bispecific or CAR T), and when in the disease course it offers the best balance between curative potential, toxicity, cost and real‐world feasibility.

## Author Contributions

**Santino Caserta**, **Mamdouh Skafi**, **Enrica Antonia Martino**, **Fortunato Morabito**, and **Massimo Gentile:** conceptualization. **Santino Caserta**, **Mamdouh Skafi**, **Enrica Antonia Martino**, **Francesco Mendicino**, **Maria Eugenia Alvaro**, **Ernesto Vigna**, **Antonella Bruzzese**, and **Fortunato Morabito:** methodology. **Enrica Antonia Martino**, **Santino Caserta**, **Fortunato Morabito**, and **Massimo Gentile:** writing – original draft preparation. **Enrica Antonia Martino**, **Santino Caserta**, **Mamdouh Skafi**, **Fortunato Morabito**, and **Massimo Gentile:** writing – review and editing. All authors have read and agreed to the published version of the manuscript.

## Funding

The authors have nothing to report.

## Ethics Statement

The authors have nothing to report.

## Conflicts of Interest

The authors declare no conflicts of interest.

## Data Availability

Data sharing not applicable to this article as no datasets were generated or analysed during the current study.
